# Prospective Immune Dynamics during the First 24 Weeks of Efavirenz Based-Antiretroviral Therapy in HIV-1-Infected Subjects, According to CD4^+^ T-Cell Counts at Presentation: The IMMUNEF Clinical Trial

**DOI:** 10.1371/journal.pone.0117118

**Published:** 2015-02-11

**Authors:** Alessandro Soria, Daria Trabattoni, Nicola Squillace, Veronica Rainone, Federica Gnudi, Mario Clerici, Andrea Gori, Alessandra Bandera

**Affiliations:** 1 Division of Infectious Diseases, Department of Internal Medicine, San Gerardo Hospital, University of Milano-Bicocca, Monza, Italy; 2 Department of Biomedical and Clinical Sciences L. Sacco, University of Milan, Milan, Italy; 3 Department of Physiopathology and Transplants, University of Milan, Milan, Italy; 4 Don Carlo Gnocchi Foundation, IRCCS, Milan, Italy; University of Rome Tor Vergata, ITALY

## Abstract

**Background:**

Longitudinal characterization of immune recovery in the first-phase of antiretroviral therapy (ART) is poorly described. We compared immune kinetics in individuals who were diagnosed early or late with HIV-1 infection, (thus commencing ART with different CD4^+^ T-cell counts), in order to investigate possible mechanisms involved in subsequent poor immune recovery.

**Methods:**

Immunophenotyping, immune activation, proliferation, apoptosis, regulatory T-cells and intracellular cytokine production were compared at baseline and during 24-week follow-up in two groups of HIV-1-infected patients initiating the same ART (tenofovir/emtricitabine/efavirenz) and divided according to baseline CD4^+^ T-cell counts (late: ≤200/μL; early: >200/μL). Wilcoxon-rank sum test and analysis for repeated measures were used to evaluate differences between groups over time.

**Results:**

Twenty-four out of 30 enrolled subjects were evaluable for the analysis, 13 late and 11 early presenters. Significantly lower CD4^+^ naïve and memory T-cells, and higher plasma viral load, as well as augmented percentages of activated (CD4^+^/CD25^+^ cells), apoptotic (CD4^+^/AnnexinV^+^/7AAD^−^, CD4^+^/caspase 8^+^ and CD4^+^/caspase 9^+^), and proliferating (CD8^+^/Ki67^+^ cells) lymphocytes were present at baseline in late presenters; ART resulted in a reduction of apoptotic and proliferating lymphocytes within the follow-up period.

**Conclusions:**

A skewing towards memory/activated/apoptotic phenotype is seen in HIV-1-infected subjects starting ART at low CD4^+^ T-cell counts; ART results in early (24 weeks) trend towards normalization of these parameters. Antiretroviral therapy may play a role in rapidly limiting aberrant immune exhaustion even in late presenters, while requiring more time for re-population of highly depleted naïve T-cells.

**Trial Registration:**

EU Clinical Trial Register EUDRACT number 2008-006188-35 https://www.clinicaltrialsregister.eu/ctr-search/trial/2008-006188-35/IT

## Introduction

Despite the effectiveness of antiretroviral therapy (ART), a remarkable percentage of HIV-1-infected patients fails to achieve satisfactory immune recovery, i.e. CD4^+^ counts >350 cells/μL or even >200 cells/μL [1]. These “immunological non responders” (INR) are at increased risk of AIDS and non-AIDS clinical events, and related mortality [2–7]. Among factors associated with INR status, some are linked to the patient’s medical history (age, hepatitis C, previous AIDS-defining events, low nadir CD4^+^ counts); others are the expression of an impaired immune system (higher immune activation and T-cell turnover, including T-cell proliferation and apoptosis) [8].

Studies aimed at identifying the mechanisms underlying this phenomenon are mainly cross-sectional. Differences in immune parameters have been found according either to different immunological response to ART (reflected by the magnitude of CD4^+^ T-cells recovery), or to different degree of immunedeficiency at presentation or during the course of HIV-1 infection (reflected by CD4^+^ T-cells nadir). These studies rarely offer a longitudinal view of what happens to the immune system in the very first months of ART, in particular comparing immune kinetics between subjects with advanced HIV-1 infection (late presenters) and those who start ART with less advanced disease (early presenters). Moreover, some peculiar aspects of immune recovery, as regulatory T-cell (Treg) homeostasis or specific anti-HIV response as measured by γ-interferon (IFN-γ) and interleukin-2 (IL-2) production, have been poorly described so far in a longitudinal view.

We designed a pilot clinical trial to prospectively explore and measure parameters related to the immune kinetics in the first 24 weeks of ART in two groups of patients divided according to their baseline CD4^+^ counts: thus, late and early presenters were compared. Our aim was to identify possible peculiarities which could increase our knowledge on mechanisms of immune reconstitution in these two different categories of HIV-1-infected patients.

In order to concentrate only on immunological characteristics and to avoid treatment-driven biases, we chose the same antiretroviral therapy for all the patients in the trial. Efavirenz was chosen as the “anchor” drug to be added to a coformulated emtricitabine/tenofovir “backbone”, as this triple therapy, since the time the protocol was designed, has been the standard of care for HIV-1-infected patients starting ART.

## Methods

The protocol for this trial and supporting TREND checklist are available as supporting information; see S1 IMMUNEF TREND Checklist and S1 IMMUNEF Protocol.

### Subjects

Consecutive ART-naïve HIV-1-infected patients presenting late or early for ART initiation (CD4^+^ ≤200 cells/μL or >200 cells/μL, respectively) received coformulated tenofovir/emtricitabine plus efavirenz, and were followed as a standard of care. Blood samples for supplementary immunological analyses were drawn at baseline, 4, 12, and 24 weeks.

Inclusion criteria (for details refer to the protocol) included age in the range of 18–50 years, the presence of a genotypic resistance test allowing the use of the study drugs, the absence of current opportunistic infections. Subjects receiving immune modulating agents (including systemic and inhaled steroids in the previous two months) were excluded.

### Ethics Statement

The study was conducted in the Division of Infectious Diseases, San Gerardo Hospital, Monza, Italy, after approval by the institutional ethics committee named “Comitato Etico Ospedale San Gerardo”. The trial was registered in the EU Clinical Trial Register, publicly available at http://clinicaltrialregister.eu, EUDRACT number 2008–006188–35. Written informed consent to participate in the study was obtained and signed by all the enrolled patients. The study was conducted according to the principles expressed in the Declaration of Helsinki.

### Immunological analyses

The following parameters were analysed both in unstimulated and in HIV peptides-stimulated peripheral blood mononuclear cells (PBMC) cultures:

- immunophenotype (naïve [CD4^+^CCR7^+^45RA^+^], central memory [CD4^+^CCR7^+^45RA^-^], effector memory [CD4^+^CCR7^-^RA^-^], terminally differentiated [CD4^+^CCR7^-^RA^+^] CD4^+^ and CD8^+^ T-cells)
- immune activation (CD8^+^CD45RO^+^CD38^+^, CD4^+^CD25^+^, CD4^+^HLA-DR^+^ T-cells)
- immune proliferation (Ki67-expressing CD4^+^ and CD8^+^ T-cells)
- apoptosis (viable, early and late apoptotic CD4^+^ T-cells; activation of caspases 8 and 9 in CD4^+^ T-cells)
- total, naïve and activated Treg (CD4^+^CD25^bright^FoxP3^+^)
- pro-inflammatory intracellular cytokine production: IL-2 and IFN-γ

Blood sample collection and peripheral blood mononuclear cell separation

Whole blood was collected by venipuncture in Vacutainer tubes containing EDTA (Becton Dickinson, Rutherford, New Jersey, USA). PBMCs were separated on lymphocyte separation medium (Cedarlane Laboratories Limited, Hornby, Ontario, Canada) and the number of viable leukocytes was determined by trypan blue exclusion test.

Stimulation of PBMC

PBMC were incubated for 18 hours in the presence/absence of a pool of gag+env peptides (HIV) (Microbics Biosystems inc., Toronto, Ontario, Canada). For cytokine analyses, 10μg/ml Brefeldin A (Sigma-Aldrich, St. Louis, Missouri, USA) was added to cell cultures during the last 6 hours.

Ki67 evaluation

PBMCs washed in PBS and stained for CD4PE mAb (Beckman-Coulter, Fullerton, California, USA) for 15 minutes at room temperature in the dark were then fixed in 1% paraformaldehyde (PFA; Sigma-Aldrich) for 15 minutes at 48°C. After washing, cells were resuspended in 0.5% saponin (Sigma-Aldrich) and stained for Ki67 or mouse fluorescein isohiocyanate (FITC)-coupled IgG1 isotype control (BD Biosciences, San Diego, California, USA). Cells were finally incubated for 45 minutes at 48°C in the dark, washed, and fixed in 1% PFA.

Identification of T-regulatory lymphocytes

PBMCs were incubated with anti-CD4, anti-CD25, and anti-PD-1 for 15 minutes at room temperature. The intracellular detection of PD-1 and FoxP3 was performed following the manufacture’s protocol (eBioscience). Intracellular or surface costaining of PD-1 and intracellular FoxP3 was performed by flow cytometry on CD4^+^CD25^bright^ gated T-cell CD4^+^ T cells/ml.

Identification of early apoptotic, late apoptotic, and necrotic cells

Stimulated PBMCs re-suspended in D-PBS (Euroclone, Siziano, Pavia, Italy) were stained with CD4, annexin V, and 7AAD monoclonal antibodies (Beckman-Coulter). After 20-minutes incubation at room temperature, cells were washed in cold D-PBS and re-suspended in D-PBS.

Detection of activated caspases 8 and 9

The FLICA Apoptosis detection kit (Immunochemistry Technologies, Bloomington, Minnesota, USA) was used to analyse caspases. FLICA reagents were prepared according to manufacturer’s instructions and added to the re-suspended cells; the mix was then incubated for 1 hour at 37°C under 5% CO_2_. After incubation, cells were washed twice with buffer. The cell pellet was re-suspended in wash buffer and stained with CD4 and CD8 monoclonal antibodies for 30 minutes in ice. Finally, cells were stained with propidium iodide and analysed by flow cytometry.

Cytometric analysis

Cytometry was performed using a FC500 flow cytometer (Beckman-Coulter) equipped with a double 15-mW argon ion laser operating at 456 and 488 nm interfaced with Intercorp computer. For each analysis 20 000 events were acquired and gated on either CD4 or CD8 expression and SSC (side scatter) properties. Green fluorescence from FITC (FL1) was collected through a 525-nm bandpass filter, orange-red fluorescence from R-PE (FL2) was collected through a 575-nm band-pass filter, Texas red fluorescence from ECD (FL3) was collected through a 613-nm band-pass filter, red fluorescence from PECy5 and APC (FL4) were collected through a 670-nm bandpass filter, far red fluorescence from PECy7 (FL5) was collected through a 770-nm band-pass filter. Data were collected using linear amplifiers for forward and SSC and logarithmic amplifiers for FL1, FL2, FL3, FL4, and FL5.

### Statistical analysis

Comparisons between the two groups of late and early presenters at different time points were performed using Wilcoxon-rank sum test. The age-adjusted analysis of variance was used to evaluate differences between the two groups (the between-patients factor) over time (the within-patient factor), applying a mixed-model repeated measures analysis, with rank-transformed variables when the distributions were not normal.

Because of the exploratory nature of the study, there was no *a-priori* evaluation of statistical power. Therefore, no correction for multiple comparisons was applied, and unadjusted *P*-values are reported.

Statistical analyses were conducted using SAS software, version 9.2 (SAS Institute Inc., Cary, NC).

## Results

### Study population

Thirty subjects signed informed consent and were enrolled in the trial between March 2009 and October 2010; four discontinued efavirenz for side-effects; one was lost to follow-up; one had virological failure at week 24, and was excluded from the analysis.

Of the remaining twenty-four subjects, (3 women, 21 men), 13 were classified as late presenters (median CD4+ T-cell counts 58 cells/μL, range 12–196 cells/μL) and 11 as early presenters (median CD4+ T-cell counts 293 cells/μL, range 224–530 cells/μL).

**Fig 1:**
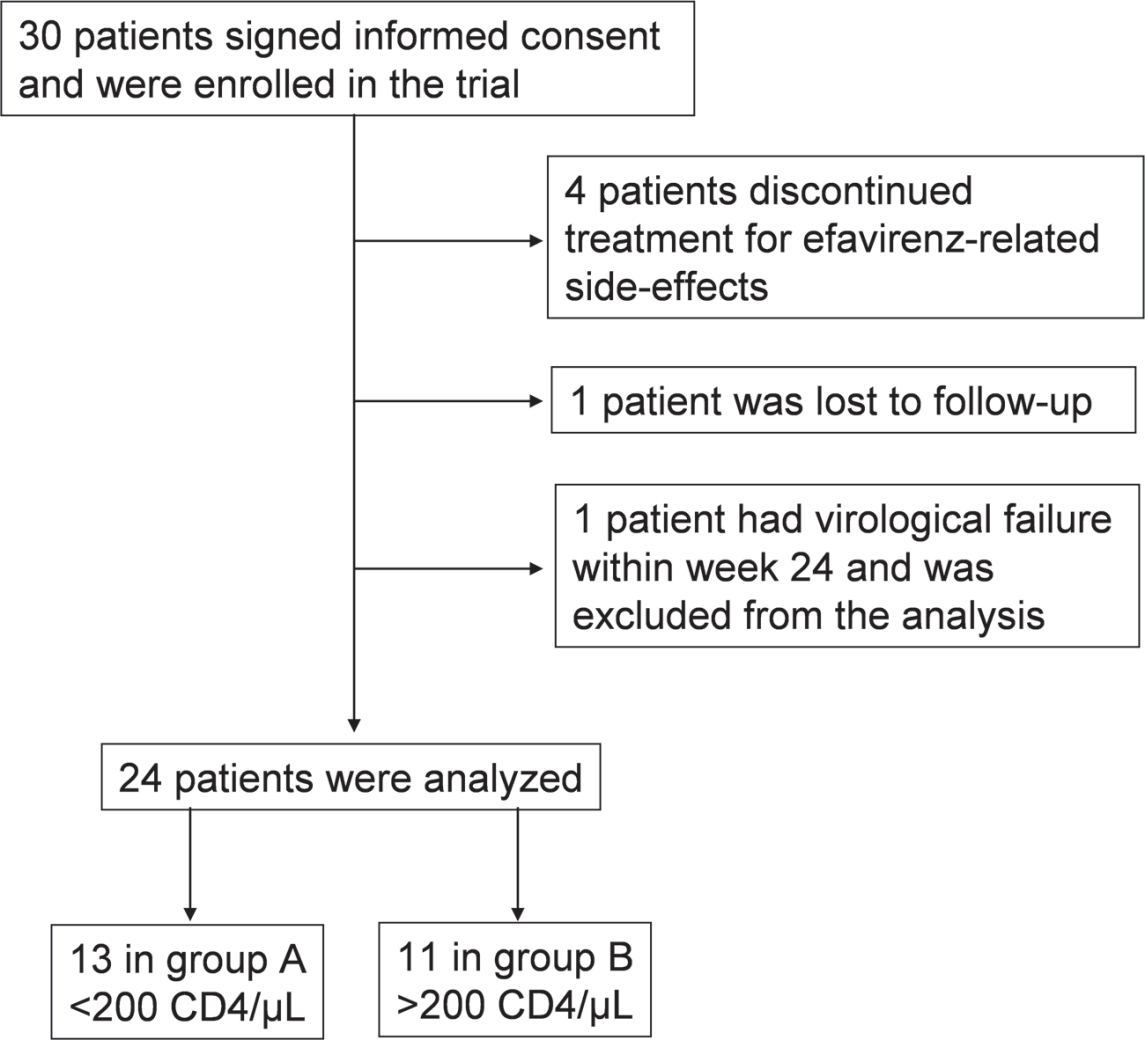
Patients’ disposition.

Patients’ disposition is shown in Fig. 1.

There were no significant differences between the two groups in the distribution of gender, route of HIV acquisition, HBV or HCV coinfection. Four late presenters had had an AIDS-defining opportunistic infection before the start of ART (S1 Table).

### Baseline differences of immune parameters

Table 1 shows baseline values of the analysed immunological parameters, stratified by group. As expected, lower values of CD4^+^ cells, CD4^+^ percentage, CD4^+^:CD8^+^ ratio, absolute CD4^+^ naïve and memory T-cells were seen in late presenters, in whom immune activation, measured by CD4^+^CD25^+^ cells, was higher as well. Notably, whereas a trend toward higher CD4^+^HLA-DR^+^ cells was seen in late presenters (p = 0.062), CD8^+^CD45RO^+^CD38^+^ cell percentages were comparable in the two groups.

**Table 1:** Demographic and immunological characteristics of the 24 HIV-1-infected treatment-naïve patients initiating their first antiretroviral therapy with tenofovir, emtricitabine and efavirenz.

	Late presenters N = 13	Early presenters N = 11	P-value
Demographic and immuno-virological characteristics			
Age (years)	44 (37–50)	39 (31–43)	0.019
Time of HIV infection (months)	1 (0–2)	22 (5–47)	0.001
Nadir CD4 (cells/μL)	58 (30–123)	293 (241–356)	<0.001
CD4 (cells/μL)	58 (30–169)	356 (282–380)	<0.001
CD4%	9.48 (7.09–11.11)	19.13 (14.21–22.01)	0.001
CD8 (cells/μL)	518 (196–1322)	1155 (838–1360)	0.079
CD8%	66.29 (53.72–75.02)	60.69 (52.51–68.07)	0.447
CD4:CD8 ratio	0.14 (0.10–0.21)	0.32 (0.21–0.44)	0.005
HIV-RNA (log_10_ copies/ml)	5.57 (5.41–5.81)	4.60 (4.41–5.03)	< 0.001
i. Immunophenotype			
CD4 naïve %	40.0 (22.6–58.2)	40.1 (30–63)	0.597
CD4 central memory %	32.1 (8.5–56.1)	32.4 (16.8–55.8)	0.972
CD4 effector memory %	13.0 (3.2–41.6)	10.2 (2.6–23.6)	0.481
CD4 terminally differentiated %	1.4 (0.0–16.0)	2.9 (0.8–7.0)	1.000
CD8 naïve %	16.0 (11.6–34.6)	21.9 (8.6–42.2)	0.860
CD8 central memory %	18.5 (6.6–25.8)	8.2 (4.7–13.4)	0.307
CD8 effector memory %	38.9 (23.3–46.6)	24.7 (11.6–53.6)	0.504
CD8 terminally differentiated %	15.2 (12.4–34.2)	20.6 (9.6–30.2)	1.000
ii. Immune activation			
CD4+CD25+ %	10.4 (6.4–17.8)	5.9 (3.4–8.0)	0.038
CD4+HLA-DR+ %	18.8 (6.4–25.0)	4.5 (3.4–9.2)	0.062
CD8+CD45RO+CD38+ %	13.5 (9.2–21.2)	14.3 (10.2–15.8)	0.916
iii. Immune proliferation			
CD4+Ki67+ %	0.7 (0.3–1.6)	0.2 (0.0–0.6)	0.171
CD8+Ki67+ %	1.6 (1.3–3.5)	0.6 (0.3–1.2)	0.034
iv. Apoptosis			
Viable CD4+ %	86.8 (77.6–90.4)	94.6 (91.5–95.1)	0.005
Early apoptotic CD4+ %	8.0 (6.2–13.0)	4.0 (3.6–7.0)	0.019
Late apoptotic CD4+ %	3.3 (1.6–7.0)	1.3 (0.8–1.6)	0.069
CD4+ Caspase 8+ %	11.2 (5.2–17.0)	3.8 (2.2–5.2)	0.004
CD4+ Caspase 9+ %	9.0 (6.6–22.6)	3.6 (2.8–5.8)	0.038
v. Regulatory T-cells (Treg)			
Total Treg %	3.1 (0.6–6.2)	0.9 (0.4–1.2)	0.139
Naïve Treg %	3.6 (2.6–11.0)	3.2 (1.2–5.0)	0.348
Activated Treg %	3.1 (2.2–10.4)	3.9 (1.8–9.4)	0.791
vi. Intracellular cytokine production			
CD4+IL-2%	1.2 (0.6–6.3)	1.1 (0.3–7.5)	0.725
CD4+IFN-γ %	2.7 (1.6–4.0)	0.8 (0.6–2.6)	0.067

The subjects are divided in two groups according to their baseline CD4^+^ counts: late presenters: ≤200 cells/μL; early presenters: >200 cells/μL. Data are expressed as medians (interquartile range, IQR).

Proliferation measured by CD8^+^Ki67^+^ cells percentage was significantly higher in late presenters; CD4^+^Ki67^+^ cells percentage was augmented as well in these patients (median 0.70% [Q1–Q3: 0.30–1.60] *versus* 0.25% [0.00–0.60]), although the difference was not statistically significant.

Apoptosis, measured by percentage of early apoptotic CD4^+^ cells, CD4^+^ caspase 8^+^ and CD4^+^ caspase 9^+^ cells, was significantly higher in late presenters. Late apoptotic CD4^+^ cells were also increased in these individuals, (median 3.30% [Q1–Q3: 1.60–7.00] *versus* 1.30% [0.80–1.60]), although the difference was not statistically significant.

Finally no significant differences were observed between the two groups when either Treg or intracellular cytokine production (IFN-γ^+^ and IL-2^+^ CD4^+^ cells) were analyzed at baseline.

### Time trends of immune parameters

Statistically significant differences observed at baseline between groups persisted over time for most of the listed variables, except for viral load and CD8^+^Ki67^+^ cells percentage.

**Fig 2:**
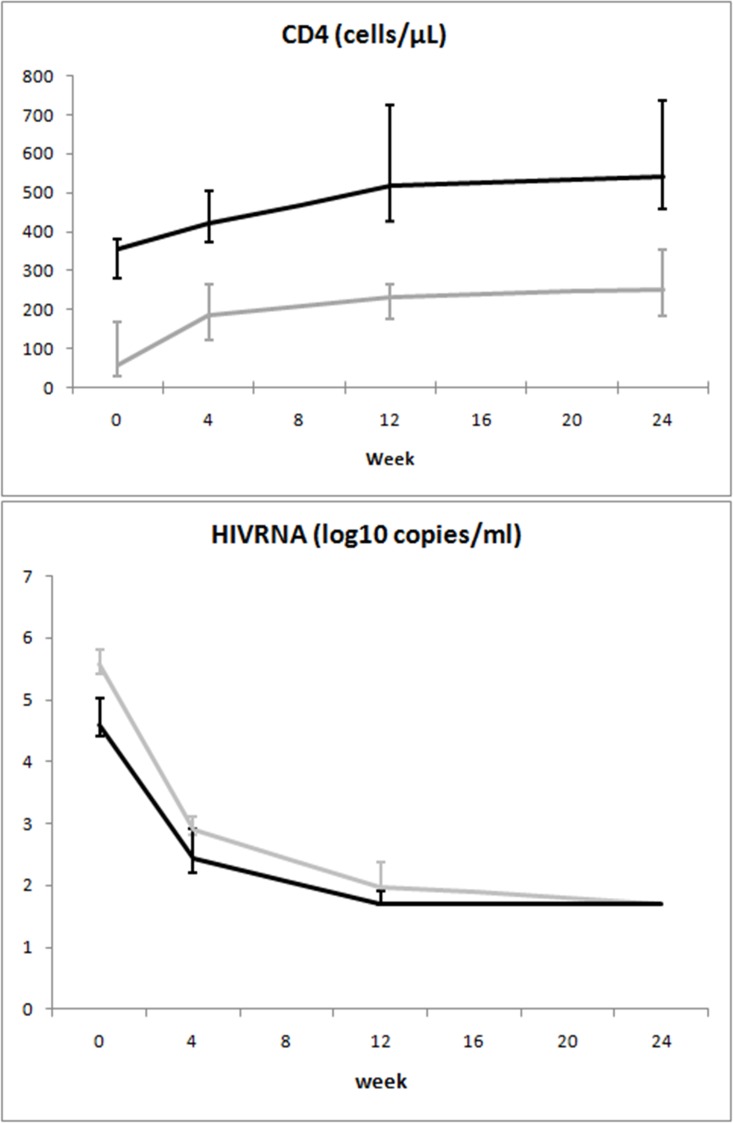
Variation over time of immunovirological parameters in two groups of HIV-1-infected patients starting their first antiretroviral treatment at different baseline CD4^+^ T-cell counts (late presenters [grey line] ≤200 cells/μL; early presenters [black line] >200 cells/μL). Medians and interquartile ranges are shown.

**Fig 3:**
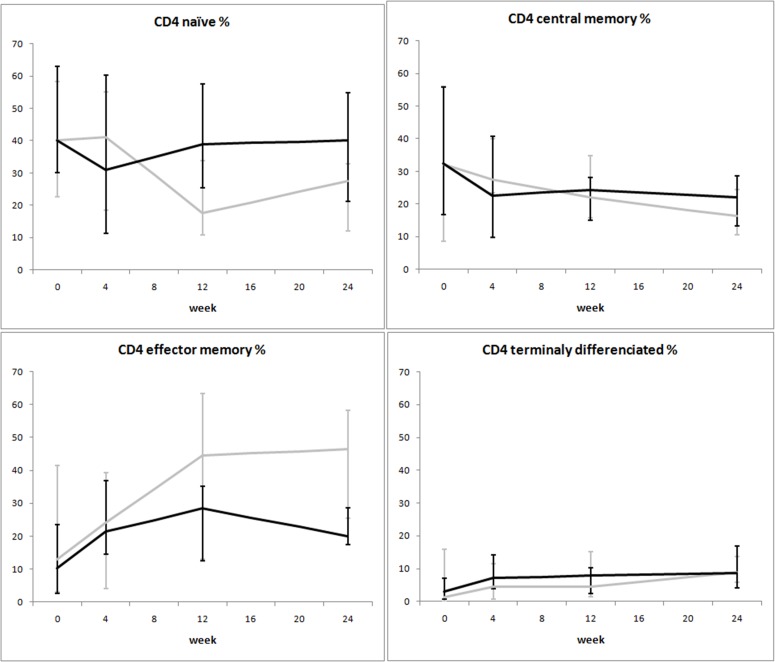
Variation over time of immunophenotype in two groups of HIV-1-infected patients starting their first antiretroviral treatment at different baseline CD4^+^ T-cell counts (late presenters [grey line] ≤200 cells/μL; early presenters [black line] >200 cells/μL). Medians and interquartile ranges are shown.

**Fig 4:**
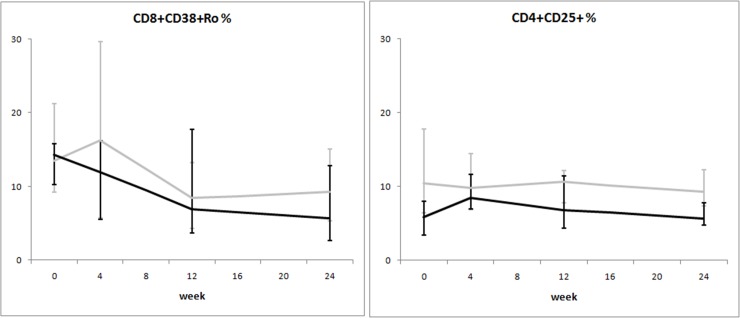
Variation over time of immune activation in two groups of HIV-1-infected patients starting their first antiretroviral treatment at different baseline CD4^+^ T-cell counts (late presenters [grey line] ≤200 cells/μL; early presenters [black line] >200 cells/μL). Medians and interquartile ranges are shown.

**Fig 5:**
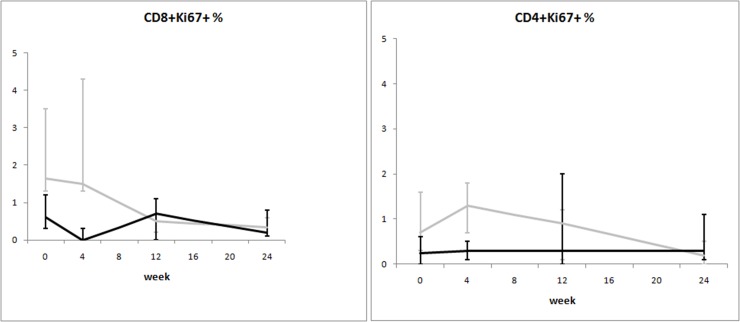
Variation over time of immune proliferation in two groups of HIV-1-infected patients starting their first antiretroviral treatment at different baseline CD4^+^ T-cell counts (late presenters [grey line] ≤200 cells/μL; early presenters [black line] >200 cells/μL). Medians and interquartile ranges are shown.

**Fig 6:**
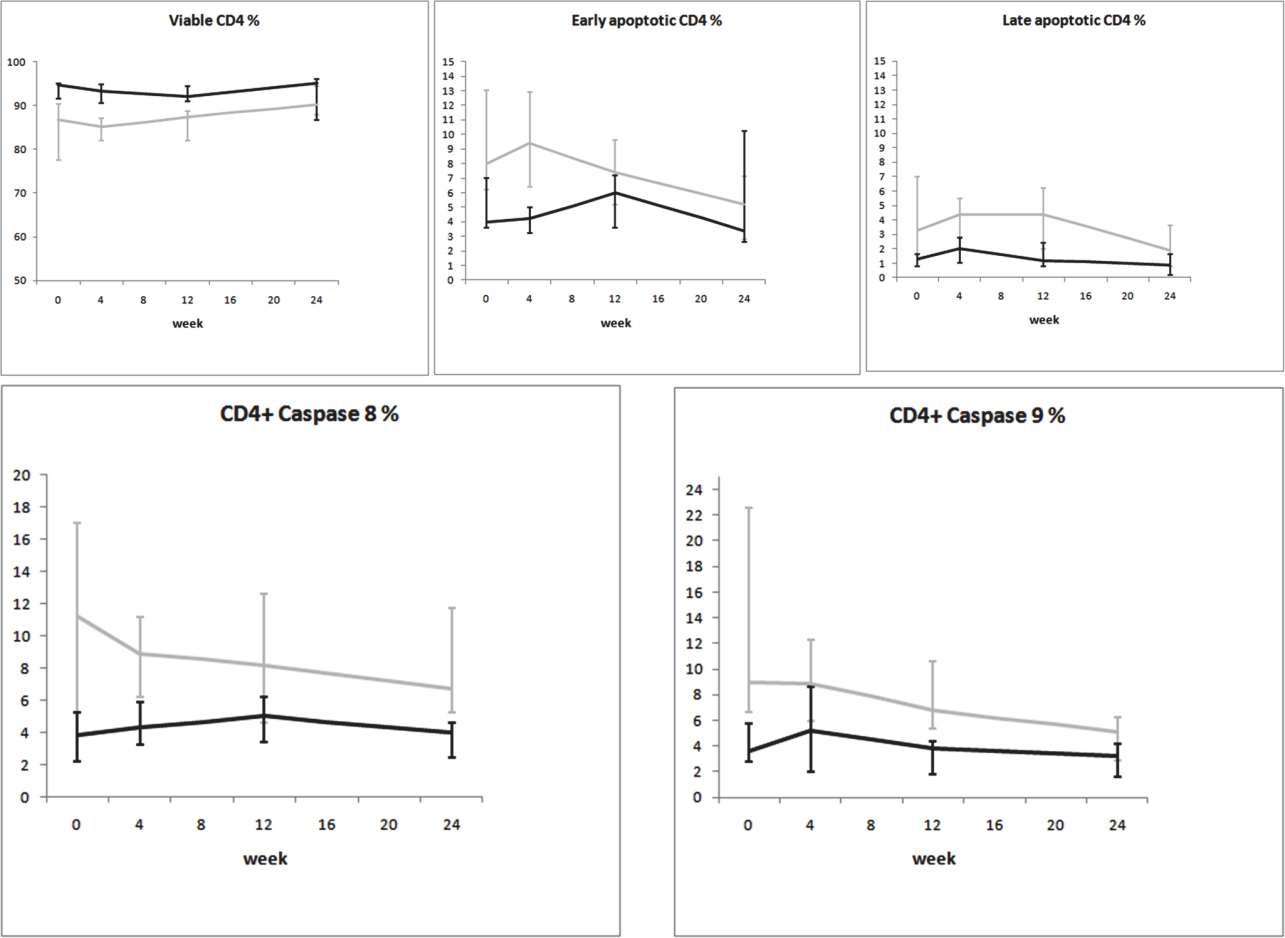
Variation over time of apoptosis in two groups of HIV-1-infected patients starting their first antiretroviral treatment at different baseline CD4^+^ T-cell counts (late presenters [grey line] ≤200 cells/μL; early presenters [black line] >200 cells/μL). Medians and interquartile ranges are shown.

**Fig 7:**
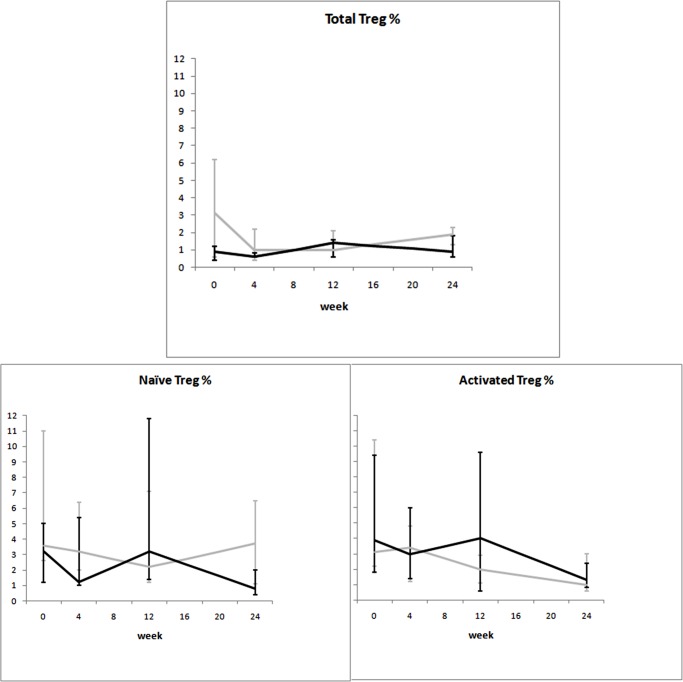
Variation over time of Treg in two groups of HIV-1-infected patients starting their first antiretroviral treatment at different baseline CD4^+^ T-cell counts (late presenters [grey line] ≤200 cells/μL; early presenters [black line] >200 cells/μL). Medians and interquartile ranges are shown.

**Fig 8:**
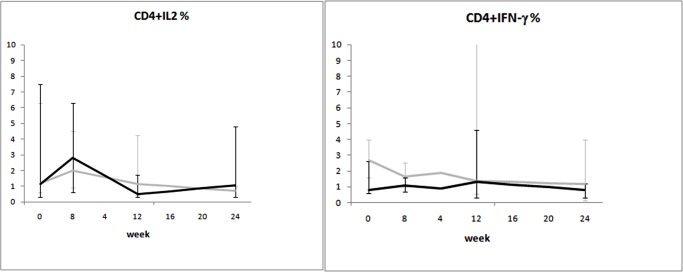
Variation over time of intracellular cytokine production in two groups of HIV-1-infected patients starting their first antiretroviral treatment at different baseline CD4^+^ T-cell counts (late presenters [grey line] ≤200 cells/μL; early presenters [black line] >200 cells/μL). Medians and interquartile ranges are shown.

When the mixed-model/repeated measures analysis was used, differences in time trends between groups were observed for viral load and CD8^+^Ki67^+^ percentage. Time trends of the considered variables are illustrated in Figs. 2–8.

Fig. 2 shows the dynamics of the “classical” immunovirological parameters: the difference in CD4^+^ counts between the two groups was maintained over 24 weeks; by contrast, undetectable viral load was achieved in both groups, regardless of a higher baseline value in patients with advanced disease.

The variation of immunophenotype in the first 24 weeks of ART was similar between the two groups (Fig. 3): the increase in the absolute number of memory CD4^+^ T-cells was mainly a consequence of an increase of effector memory CD4^+^ rather than central memory CD4^+^ T-cells. Of note, increases in effector memory CD4^+^ T cells were more pronounced in the subjects with low baseline CD4^+^ counts, even if not statistically significant. By contrast, no evident gain in naïve CD4^+^ cells was observed in the first 24 weeks in both groups.

Overall, immune activation parameters at the end of the follow up period were higher in late presenters (Fig. 4). Notably, CD8^+^CD45RO^+^CD38^+^ cells, which were similar at baseline between the two groups, diminished over time more markedly after initiation of ART in early presenters, reaching a lower level at week 24 as compared to late presenters (median 5.7% *versus* 9.2%).

The dynamics of immune proliferation markers between the two groups is shown in Fig. 5. Higher percentages of CD8^+^Ki67^+^ cells were seen at baseline in late presenters: this difference disappeared over time, as these cells became comparable between the two groups after 24 weeks of ART. Time trends for markers of apoptosis are shown in Fig. 6. Late presenters show a general trend of decrease over time (except for viable CD4^+^ cells), whereas no marked trends emerged for early presenters (even if differences between groups are not statistically significant).

Finally, no consistent trend over time was observed for either Treg dynamics (Fig. 7) or cytokine production (IL-2 and IFN-γ) (Fig. 8).

## Discussion

The increase in CD4^+^ counts during ART is highly variable among HIV-1-infected patients. Our prospective pilot study aimed at identifying possible differences in immune reconstitution between subjects initiating treatment in advanced stage of HIV disease, compared with those starting therapy at around 350 CD4^+^ cells/μL.

Not surprisingly, absolute counts of naïve CD4^+^ cells were lower in patients with advanced disease. This difference was maintained throughout 24 weeks of treatment, confirming that, once ART is initiated, more time is needed for the *de novo* synthesis of CD4^+^ cells. By contrast, the increase in memory CD4^+^ cells, mainly effector memory rather than central memory, reflects the known “redistribution effect” from lymphoid organs to peripheral blood of the first phase of immune reconstitution.

Confirming previous literature [8–11], we found higher levels of immune activation, immune proliferation, and apoptosis in treatment-naïve patients with low CD4^+^ counts. Our results did not change after adjusting for age, a variable recently found to be a strong confounder in determining the levels of immune activation [12]. This is not surprising, as we deliberately excluded patients over 50 years of age, in order to avoid excessive age influence on immune activation outcome. Antiretroviral therapy likely hampered the accelerated T-cell turnover that leads to immune exhaustion. This resulted in reduced immune activation, immune proliferation, and apoptosis in all patients over time. Of note, we observed differences in the kinetics of these parameters between the two groups. Specifically, while immune activation was still higher after 24 weeks of treatment in patients starting ART at low CD4^+^ counts (Fig. 4), immune proliferation and apoptosis were rapidly reduced in these same individuals, reaching approximately the same level seen in subjects who started ART at 350 CD4^+^ cells/μL (Fig. 5 and Fig. 6).

Despite the fact that the small sample size we analyzed and the wide IQRs of many analyzed variables do not allow to draw definitive conclusions, to our knowledge few studies have addressed the longitudinal variation of functional and qualitative parameters of immune recovery to ART.

Our results suggest that, even if the variation of immune parameters in the very first period of ART follows a different kinetics in patients starting treatment with low CD4^+^ counts, ART results in a rapid normalization of these parameters, with the exception of the replenishment of naïve T-cells, in both groups of individuals. The clinical implication stemming from our results is that even in late presenter there might be the possibility to rapidly limit aberrant immune exhaustion, thus achieving a satisfactory “qualitative” immune restoration. Although a sustained benefit of an earlier initiation of therapy might involve quantitative immune parameters, the effect of ART on qualitative immune features seems to be at least partially independent from CD4^+^ counts. The immediate initiation of ART therapy in patients with advanced disease could in fact result in a rapid improvement of abnormalities related to defects in proliferation and apoptosis and, even if there is not a rapid response in terms of increase in CD4^+^ T-cell count, a significant improvement in the quality of immune response, which clinically may account for the improved survival observed in patients affected by full-blown AIDS and opportunistic infections, once they start receiving ART.

Conversely, the persistence of CD8^+^ T-cell immune activation might result in a higher incidence of non-AIDS related events (a broad spectrum of cardiovascular, renal, neurocognitive events, and non-AIDS malignancies), that are more common in the lifespan of patients starting ART at low CD4^+^ T-cell counts, despite effective virologic control and immune recovery. Timely initiation of ART (before profound CD4^+^ T-cell depletion occurs) could at least in part prevent irreversible immune dysfunction, and reducing persistent immune activation might possibly limit the occurrence of non-AIDS clinical events in HIV-infected people [13–15].

In conclusion, the initiation of ART in HIV-1-infected individuals with profound immunedeficiency, through the rapid decrease of immune proliferation and apoptosis parameters, could limit immune exhaustion and account for stopping AIDS progression; on the other side, the persistence of immune activation despite effective ART could account for higher occurrence of non-AIDS-related complications on the long term.

## Supporting Information

S1 IMMUNEF Protocol (DOC)

S1 IMMUNEF TREND Checklist (PDF)

S1 Table Baseline characteristics (categorical variables) of late and early presenters in the IMMUNEF study. (DOC)
