# Supplementary material for: Prospective Immune Dynamics during the First 24 Weeks of Efavirenz Based-Antiretroviral Therapy in HIV-1-Infected Subjects, According to CD4+ T-Cell Counts at Presentation: The IMMUNEF Clinical Trial
Source: PLoS One. 2015 Feb 11;10(2):e0117118. doi: 10.1371/journal.pone.0117118 (PMC4324909; doi:10.1371/journal.pone.0117118)
Supplement: S1 IMMUNEF Protocol — (DOC) [file pone.0117118.s001.doc]

**IMMUNOLOGICAL EFFICACY OF EFAVIRENZ-BASED TREATMENT IN HIV-POSITIVE NAÏVE PATIENTS (A PYLOT STUDY)**

**CODICE PROTOCOLLO: IMMUNEF**

**CODICE EUDRACT: 2008-006188-35**

Principle Investigator: Andrea Gori, MD

Affiliation: Unit of Infectious Diseases, A.O. “S. Gerardo”, Monza

Signature of Investigator:


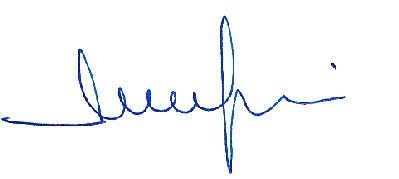


Date: 14 March 2008

## TABLE OF CONTENTS

**SUMMARY OF THE STUDY** 4

**1. STUDY BACKGROUND/RATIONALE** 8

**2. OBJECTIVE OF THE STUDY** 10

2.1 Primary objectives 10

2.2 Secondary objectives 10

**3**. **END POINTS** 10

3.1 Primary end-points 10

3.2 Secondary end-points 10

**4. STUDY DESIGN** 10

**5. METHODOLOGY** 11

5.1.1 Inclusion and eclusion criteria 11

5.2 Treatment 12

5.2.1 Study drugs 12

5.3 Treatment design 12

5.3.1 Dosing schedules 12

5.3.2 Treatment discontinuation 12

5.3.3 Dosing, time of treatment, drug administration 12

5.3.4 Dosing compliance 12

5.4 Concomitant therapy 13

5.4.1 Allowed concomitant therapy 13

5.4.2 Unallowed concomitant therapy 13

**6. ADVERSE EVENTS**  13

6.1 Definition 13

6.2 Reporting of adverse events 13

6.3 Serious adverse event 13

6.4 Efavirenz-related adverse events 13

**7. CLINICAL AND LABORATORY ASSESSMENTS** 14

7.1 Screening/Baseline Evaluations 14

7.2 Clinical evaluation 14

7.3 Laboratory tests 14

7.4 Immunology 14

7.5 Virology 16

**8. DATA ANALYSIS** 16

**9. WITHDRAWING FROM THE STUDY** 16

**10. ETHICS**  16

10.1 Declaration of Helsinki and ethics committee 16

10.2 Subject information and informed consent 16

**11. SOURCE DATA AND CASE REPORT FORMS**  17

**REFERENCES** 18

**SUMMARY OF THE STUDY**

**Rationale**

The widespread use of highly active antiretroviral therapy (HAART) is associated with significant immune reconstitution and reduction in mortality in HIV-1 infected patients. However, patients presenting for care with very low CD4 counts (so called advanced naïve) have been shown to display a slower and less efficient immune recovery, despite the prompt institution of the most potent antiretroviral treatments [1].

Besides well-defined virologic efficacy, an initial efavirenz (EFV)-based HAART regimen in HIV-infected antiretroviral-naïve patients with any CD4 counts has consistently associated to a beneficial immunological efficacy; the receipt of EFV regimen resulted in an immunologic advantage consisting of ongoing long-term immune reconstitution [2-6].

On the basis of these data, we hypothesise that the immune recovery kinetics in patients receiving an EFV-based HAART is similar in advanced (CD4 50-150 cells/μl) and less advanced (CD4>250 cells/μl) naïve subjects.

**Study description**

This is a prospective, phase IV, multicenter, open label study to evaluate the immunological efficacy of efavirenz-based antiretroviral treatment in HIV-infected patients with advanced state of immune depression, CD4+ cell count between 50/μL and 150/μl, naïve to antiretroviral therapy, compared to subjects who start antiretroviral therapy with CD4+ cell count higher than 250/μl.

**Objectives**

Primary objectives

1. To describe and compare the kinetics of immune recovery degree and quality in advanced naïve HIV-infected individuals (CD4+ 50-150 cells/μl) starting as first regimen an EFV-based HAART regimen compared to “less advanced” patients (CD4+>250 cells/μl)
2. To thoroughly characterise the role and kinetics of specific immune parameters in immune reconstitution following EFV-based HAART

Secondary objectives

1. To describe the kinetics of HIV-RNA decay in plasma of patients of the two groups
2. To describe the kinetics of intracellular HIV DNA in total peripheral blood CD4

**Endpoints**

Primary end-points

The primary efficacy variables of the study are:

- proportion of subjects who have a CD4+ T cell number increase > 20% after 24 weeks of EFV-based HAART;
- changes from baseline of peripheral CD4+ and CD8+ T-cell activation and proliferation rates;
- changes from baseline of peripheral CD4+ and CD8+ apoptosis rate;
- changes from baseline of levels and function of interleukin-7 (IL-7) and interleukin-15 (IL-5), as T-cell homeostasis cytokine regulators;
- frequency of circulating HIV-specific IFN-γ and IL-2 producing T-cells;
- frequency of circulating HIV-specific CD8+ IFN-γ ELISPOT responses;
- frequency of circulating T-regulatory CD4+ T cells (Treg);
- changes from baseline of patterns of CD4 and CD8 T-cell maturation.

Secondary end-points

- number of patients with HIV-RNA<50 cp/ml and with HIV-RNA< 5 cp/ml after 12 and 24 weeks of EFV-based HAART;
- changes form baseline of intracellular HIV DNA in total peripheral blood CD4 T cells;
- number of patients who develop treatment-induced adverse events
- number of patients who develop HIV-related clinical events

**Study design**

Prospective, phase IV, multicenter, controlled, open label study.

15 consecutive patients, responding to the inclusion/exclusion criteria, will enter each arm of the study, for a total of 30 patients.

At screening visit, patients will be assessed for demographic characteristics, and eventual HIV history. Blood tests will be performed (cell count, hematochimic tests, HIV-RNA, CD4, genotype). At baseline visit, patients will start therapy with respect of inclusion/exclusion criteria.

Blood samples will be collected at Day 1, Month 1, Month 3, Month 6.

Only for the viral decay study, samples will be collected, on top the scheduled visits, at Day 7, Day 15 and Day 45.

**Treatment administration**

After the enrolment, patients will start antiretroviral treatment with efavirenz 600 mg QD plus a fixed double NRTI-backbone, composed of tenofovir disoproxol fumarate and emtricitabine in fixed dose formulation OD.

During the study, antiinfective primary and secondary prophylaxis will be administered according to patients’ clinical and immunological parameters.

**Casistic**

Patients enrolment will be carried out in a multicenter HIV cohort group coordinated by the Unit of Infectious Diseases, San Gerardo Hospital, Milano-Bicocca University, Monza, and including the Unit of Infectious Diseases, Busto Arsizio Hospital, the Department of Infectious Diseases Luigi Sacco Hospital, Milan, and the Clinic of Infectious Diseases, S. Martino Hospital, Genova.

30 patients will be included in the trial:

Arm 1: advanced naïve, HIV-positive patients with CD4+ T cell count between 50 and 150/μl, independently of HIV-viremia levels, and naïve to antiretroviral treatments: 15 patients;

Arm 2: less advanced naïve, HIV-positive patients with CD4+ T cell count > 250/μl, independently of HIV-viremia levels, and naïve to antiretroviral treatments: 15 patients.

Inclusion criteria:

- male or female subject 18 to 50 years of age inclusive;
- the subject should be able to understand and comply with protocol requirements, instruction and protocol-stated restrictions;
- signed and dated written informed consent form prior to admission to the study;
- HIV positive status tested with ELISA and confirmed by Western Blot;
- documented CD4+ T cell count between 50 and 150/μl (advanced)
- documented CD4+ T cell count > 250/μl (less advanced);
- genotypic test allows study drugs;
- no prior or current use of antiretroviral therapy;
- no current opportunistic infection (at least 2 weeks must have passed from the end of the therapy for OI);
- no current use of systemic or inhaled steroids within the prior two months;
- no current use of immune modulating agents, HIV immunotherapeutic agents or vaccines;
- if female, be neither pregnant nor breastfeeding, and willing to use two methods of contraception (physical and hormonal double barrier method) throughout the duration of the study or be menopausal or of non-child bearing potential. All subjects should have been advised on practice of safe sex.

Exclusion criteria:

- in the investigator’s opinion subject who is unlikely to comply with the protocol and complete the study;
- primary HIV-1 infection;
- current opportunistic infection;
- genotypic test not allowing the study drugs;
- subject with current abuse of alcohol (>21 units/week or >3 units/day for males; >14 units/week or >2 units/day for females [1 unit is equivalent to half-pint (220 ml) of beer or 1 (25 ml) measure of spirits or 1 glass (125 ml) of wine] or illicit drug use;
- current use of immune modulating agents or HIV immunotherapeutic vaccines;
- current use of natural or herbal therapies;
- current use of systemic or inhaled steroids and/or use of these therapies within the two months prior to enrolment;
- pregnant or lactating female;
- subject with any of the following laboratory results within 14 days prior to the first dose of the antiretroviral treatment:
- haemoglobin concentration < 10 mg/dl for men and < 9.0 mg/dl for women;
- neutrophil count < 1000 cells/mm^3^;
- platelet count < 75,000 cells/mm^3^;
- AST and ALT > 2.5 times upper to the normal value;
- serum creatinin > 2 times upper to the normal value;

- Karnofsky index < 50.

**Methodology of the study**

Clinical evaluation

At screening visit and at Month 1, Month 3, Month 6:

- medical history
- physical evaluation
- Karnofsky index evaluation.

Haematology and clinical chemistry

At screening visit and at Day 1, Month 1, Month 3, Month 6:

- haematological complete examination with leucocytes count;
- clinical chemistry: glycemia, urea, creatinine, sodium, potassium, chloride, carbonate, AST, ALT, CPK, total bilirubin, alcaline phosphatase, pancreatic amylases, total cholesterol, triglycerides, LDH, seroproteic electrophoresis, VES
- urine β-HGC at enrolment.

# Immunological evaluation

At screening visit and at Day 1, Month 1, Month 3, Month 6:

- lymphocyte immunephenotyping:
- CD4 *memory* (CD45RA-CD62L-)
- CD4 *naïve* (CD45RA+CD62L+)
- activated CD4/CD8 (CD38+HLADR+)
- pre-terminally differentiated CD8 (CCR7- CD45RA-)
- terminally differentiated CD8 (CCR7- CD45RA+)
- effector CD8 (CD27- Perforin +)
- CD4/CD8 proliferation antigen (Ki67)
- CD4/CD8 IL-7 receptor expression (CD127)
- CD4+ T-cell apoptosis (Annexin incorporation)
- IL-7 plasma levels;
- IL-7 production;
- IL-7Rα production;
- HIV-specific CD4 lymphocytes (number, cytokine production [IFN-γ and IL-2], maturation pattern [expression of CD45RA, CCR7], receptor expression [IL-2Rα or CD25, IL-15Rα]);
- HIV-specific CD8+ lymphocytes (number, IFN-g and perforin production, maturation pattern [expression of CD45RA, CCR7], receptor expression (IL-2Rα or CD25, IL-15Rα);
- Treg lymphocytes (CD4^+^CD25^++^Foxp3^+^, number and grade of maturation [expression of CD62L]).

Virological evaluation

At screening visit and at Day 1, Day 7, Day 15, Day 45, Month 1, Month 3, Month 6:

- evaluation of intracellular HIV-DNA in total peripheral blood CD4+;
- evaluation of HIV-RNA using 2 different assays: a classical assay with a lower limit of detection of 50 cp/ml and an ultrasensitive assay with a detection limit of 5 cp/ml.

**1. STUDY BACKGROUND/RATIONALE**

Human immunodeficiency virus 1 (HIV-1) infection is characterized by a progressive decline in both function and number of CD4+ T-lymphocytes secondary to ongoing viral replication. Without intervention, this ultimately leads to the development of the Acquired Immunodeficiency Syndrome (AIDS) that places persons at risk for the acquisition of opportunistic infections and neoplasms. In recent years, reconstitution of the immune system of HIV-1-infected patients has been achieved by suppression of HIV-1 replication through antiretroviral therapies (ART), resulting in a dramatic decline in HIV-1-related morbidity and mortality [7,8].

Whereas in the short term, restoration of numbers of circulating CD4+ T-cells seems to largely protect persons from opportunistic infections, it is less clear that functional immune responses can be fully restored particularly in persons with advanced stages of HIV infection (so called advanced naïve) [1]. Recent findings from clinical trials and epidemiological studies suggest that the timing of treatment initiation is a major determinant of the capacity of the immune system for reconstitution. Findings from these studies indicate that immune phenotype and function remain impaired over time in patients who initiate ART at lower CD4+ T-cell counts even if circulating CD4+ T-cell numbers are normalized.

Despite many attempts to increase the uptake of HIV testing worldwide, many individuals are still not diagnosed until their CD4 cell count has already fallen to below 200 cells/μl [8]. The implications of a low CD4 cell count are widespread: as well as being at higher risk of clinical events while the CD4 cell count is low [9, 10], those who start HAART with very low CD4 cell counts appear to be less likely to have a sustained virological response compared with those starting at higher CD4 cell counts [11, 12]. Patients presenting for care with very low CD4 cell counts may make an overall large demand on clinical resources, thus representing a relevant challenge for HIV/AIDS clinicians.

Data from HIV+, antiretroviral patients with any CD4 count receiving EFV regimens consistently proved a prompt immunological efficacy [2-6]; the receipt of EFV regimen resulted in an immunologic advantage consisting of ongoing long-term immune reconstitution. However, few data exist thus far that thoroughly and selectively focus on the detailed time course and features of the immune reconstitution pattern following initiation of a EFV-based HAART regimen in advanced naïve patients. Indeed, while the actual efficacy of HAART in broadly restoring the specific HIV-driven “immunological wholes” in terms of both homeostasis and function is still somehow confused, this seems even more true and dramatic in the context of advanced HIV infection.

The hallmark of HIV infection is the progressive depletion of CD4+ T-lymphocytes, associated to profound qualitative immune alterations. However, the nature and source of the selective CD4+ depletion and the characterization of the viro-immunological dynamics of HIV-driven immunedeficiency still remain controversial. Direct viral citopathogenicity alone does not provide enough reason for the HIV-driven immunedeficiency. Instead, a more complex model which also includes T-cell dynamics and homeostatic regulation as the central determinants behind HIV pathogenesis, that affects several aspects of the infection, is now favoured. The physiologic maintenance of CD4+ T-cell counts in the periphery results from the equilibrium among cell depletion, redistribution, and T-cell repopulation. HIV infection is characterised by a profound state of T-cell immune hyperactivation, associated to high levels of peripheral T-cell proliferation. Such an hyperactivated/hyperproliferating immune status has been considered as one of the main determinants of HIV-driven CD4+ T-cell depletion, thus favouring T-cell sequestration within lymphoid organs and programmes T-cell death. A more comprehensive evaluation of T-cell dynamics and homeostasis has to take into account the investigation of interleukin-7 (IL-7)/IL-7R system, given the central role of IL-7 in T-cell homeostasis and function regulation.

From a functional standpoint, HIV infection is featured by the selective loss of both HIV- specific CD4 T-helper and CD8 CTL function [13]. In particular, HIV-specific CD4 display a specific lack of IL-2 production, which is also associated to a maturational block, and a relative outgrowth of IFN-γ-producing CD4 [14, 15]. An identical maturational defect have been shown to feature the CD8 pool, also displaying a reduced proliferative capacity and perforin production.

Lots of attention has been recently paid to a population of regulatory T-lymphocytes (Treg) which have been attributed a major role in the regulation of T-cell homeostasis and function. This T-cell subset constitute up to 5% of peripheral CD4, and *in vivo* display a suppressive effect on T-cells, thus playing a major role on the immune response against infections [15]. In the course of HIV infection, Treg have been shown to redistribute from peripheral blood to secondary lymphoid organs, thus suggesting that ongoing HIV viral replication is able to induce Treg tissue redistribution, in turn responsible of the alteration in immune function.

We thus aimed to assess the impact of an EFV-based HAART regimen in advanced naïve, severely immunocompromised HIV-infected patients in terms of immune recovery kinetics and degree. The deepest comprehension of the immunologic pathways behind HAART-driven immune reconstitution could indeed turn out extremely useful to better design tailored antiretroviral regimens for these critical patients.

**2. OBJECTIVES OF THE STUDY**

**2.1 Primary objectives:**

- To describe and compare the kinetics of immune recovery degree and quality in advanced naïve HIV-infected individuals (CD4+ 50-150/μl) starting as first regimen an EFV-based HAART regimen compared to “less advanced” (CD4+ >250/μl)

- To thoroughly characterise the role and kinetics of specific immune parameters in immune reconstitution following EFV-based HAART

**2.2 Secondary objectives:**

- To describe the kinetics of HIV-RNA decay in plasma of patients of the two groups
- To describe the kinetics of intracellular HIV DNA in total peripheral blood CD4

##### 3. END POINTS

**3.1 Primary end-points:**

The primary efficacy variables of the study are:

- proportion of subjects who have a CD4+ T cell number increase > 20% after 24 weeks of EFV-based HAART;
- changes from baseline of peripheral CD4+ and CD8+ T-cell activation and proliferation rates;
- changes from baseline of peripheral CD4+ and CD8+ apoptosis rate;
- changes from baseline of levels and function of interleukin-7 (IL-7) and interleukin-15 (IL-5), as T-cell homeostasis cytokine regulators;
- frequency of circulating HIV-specific IFN-γ and IL-2 producing T-cells;
- frequency of circulating HIV-specific CD8+ IFN-γ ELISPOT responses;
- frequency of circulating T-regulatory CD4+ T cells (Treg);
- changes from baseline of patterns of CD4 and CD8 T-cell maturation.

**3.2 Secondary end-points**:

- number of patients with HIV-RNA<50 cp/ml and with HIV-RNA< 5 cp/ml after 12 and 24 weeks of EFV-based HAART;
- changes form baseline of intracellular HIV DNA in total peripheral blood CD4 T cells;
- number of patients who develop treatment-induced adverse events
- number of patients who develop HIV-related clinical events.

**4. STUDY DESIGN**

Prospective, phase IV, multicenter, controlled, open label study.

15 consecutive patients, responding to the inclusion/exclusion criteria, will enter each arm of the study, for a total of 30 patients.

At screening visit, patients will be assessed for demographic characteristics, and eventual HIV history. Blood tests will be performed (cell count, haematochimic tests, HIV-RNA, CD4, genotype). At baseline visit, patients will start therapy with respect of inclusion/exclusion criteria.

Blood samples will be collected at Day 1, Month 1, Month 3, Month 6.

Only for the viral decay study, samples will be collected, on top the scheduled visits, at Day 7, Day 15 and Day 45.

**Casistic**

Patients enrolment will be carried out in a multicenter HIV cohort group coordinated by the Unit of Infectious Diseases, San Gerardo Hospital, Milano-Bicocca University, Monza, and including the Unit of Infectious Diseases, Busto Arsizio Hospital, the Department of Infectious Diseases Luigi Sacco Hospital, Milan, and the Clinic of Infectious Diseases, S. Martino Hospital, Genova.

30 patients will be included in the trial:

Arm 1: advanced naïve, HIV-positive patients with CD4+ T cell count between 50 and 150/μl, independently of HIV-viremia levels, and naive to antiretroviral treatments: 15 patients;

Arm 2: less advanced naïve, HIV-positive patients with CD4+ T cell count > 250/μl, independently of HIV-viremia levels, and naive to antiretroviral treatments.

After the enrolment, patients will start antiretroviral treatment with efavirenz plus a fixed double NRTI-backbone, composed of tenofovir disoproxol fumarate and emtricitabine.

The length of the study will be 24 weeks (6 months).

**5. METHODOLOGY**

**5.1 Inclusion and exclusion criteria**

Inclusion criteria:

- male or female subject 18 to 50 years of age inclusive;
- the subject should be able to understand and comply with protocol requirements, instruction and protocol-stated restrictions;
- signed and dated written informed consent form prior to admission to the study;
- HIV positive status tested with ELISA and confirmed by Western Blot;
- documented CD4+ T cell count between 50 and 150/μl;
- documented CD4+ T cell count >250/μl;
- no prior or current use of antiretroviral therapy;
- no current opportunistic infection (at least 2 weeks must have passed from the end of the therapy for OI);
- no current use of systemic or inhaled steroids within the prior two months;
- no current use of immune modulating agents, HIV immunotherapeutic agents or vaccines;
- if female, be neither pregnant nor breastfeeding, and willing to use two methods of contraception (physical and hormonal double barrier method) throughout the duration of the study or be menopausal or of non-child bearing potential. All subjects should have been advised on practice of safe sex.

Exclusion criteria:

- in the investigator’s opinion subject who is unlikely to comply with the protocol and complete the study;
- primary HIV-1 infection;
- current opportunistic infection;
- genotypic test not allowing the study drugs;
- subject with current abuse of alcohol (>21 units/week or >3 units/day for males; >14 units/week or >2 units/day for females [1 unit is equivalent to half-pint (220 ml) of beer or 1 (25 ml) measure of spirits or 1 glass (125 ml) of wine] or illicit drug use;
- current use of immune modulating agents or HIV immunotherapeutic vaccines;
- current use of natural or herbal therapies;
- current use of systemic or inhaled steroids and/or use of these therapies within the two months prior to enrolment;
- pregnant or lactating female;
- subject with any of the following laboratory results within 14 days prior to the first dose of the antiretroviral treatment:
- haemoglobin concentration < 10 mg/dl for men and < 9.0 mg/dl for women;
- neutrophil count < 1000 cells/mm^3^;
- platelet count < 75,000 cells/mm^3^;
- AST and ALT > 2.5 times upper to the normal value;
- serum creatinin > 2 times upper to the normal value;

- Karnofsky index < 50.

**5.2 Treatment**

5.2.1 Study drugs:

Study drug was defined as efavirenz (Sustiva®), available as 600 mg tablets.

Dual-NRTI backbone therapy will be composed of tenofovir disoproxol fumarate (TDF) and emtricitabine (FTC) in fixed dose formulation OD.

**5.3 Treatment design**

5.3.1 Dosing schedules:

Arm 1: TDF+FTC + efavirenz for 6 month according to conventional dosing.

Arm 2: TDF+FTC + efavirenz for 6 month according to conventional dosing.

5.3.2 Treatment discontinuation:

Subjects must be withdrawn from the trial if:

- the subject experiences grade 3 or 4 adverse events;

- the subject experiences serious adverse events in physician’s consideration.

Subjects may be withdrawn from the trial if:

- the investigator considers it is the best interest to the subject, for safety reason, that he or she be withdrawn;
- the subject fails to comply with the protocol or study staff requirements.

5.3.3 Dosing, time of treatment, drug administration:

Nucleoside reverse transcriptase inhibitors (NRTIs) and efavirenz will be administered according to conventional dosing. Dose modifications should be applied in case of drug interactions with concomitant medications according to DHHS Antiretroviral guidelines.

The actual scheme of antiretroviral combination should be carried on for at least 6 months.

5.3.3 Dosing compliance:

Any unscheduled dosing modifications should be reported and justified in clinical records.

**5.4 Concomitant therapy**

5.4.1 Allowed concomitant therapy:

Therapies considered necessary to patients health (e.g. antiinfective primary and secondary prophylaxis or other antiviral and antibacterial treatments) will be administered according to patients’ clinical and immunological parameters.

Any concomitant therapy must be reported in the clinical record.

### 5.4.2 Disallowed concomitant therapy:

### Use of immune modulating agents or HIV immunotherapeutic vaccines.

**6. ADVERSE EVENTS**

The patients should relate to the investigator any adverse event.

**6.1 Definition**

Any untoward medical occurrence in a subject or clinical investigation subjects administered a pharmaceutical product and which dose not necessarily have a casual relationship with this treatment. An AE can therefore be any unfavourable and unintended sign (including an abnormal laboratory finding), symptom, or disease temporally associated with the use of a medicinal (investigational) product, whether or not related to the medicinal (investigational) product.

**6.2 Reporting of adverse events**

Adverse events will be classified according to the WHO definition. All adverse events must be reported in the clinical record, whether or not related to the investigational product.

All AEs leading to discontinuation or treatment interruption and still ongoing at the end of the treatment will be followed until satisfactory resolution or stabilization.

**6.3 Serious adverse event**

Any untoward medical occurrence that at any dose:

- results in death;

- is life threatening;

- requires inpatient hospitalization or prolongation of existing hospitalization;

- results in persistent or significant disability/incapacity or

-is a congenital anomaly/birth defect.

**6.4 Efavirenz-related adverse events**

The most significant adverse events observed in patients treated with efavirenz are nervous system symptoms, psychiatric symptoms, and rash. Nervous system symptoms include: [dizziness](http://www.rxlist.com/script/main/art.asp?articlekey=6114), [insomnia](http://www.rxlist.com/script/main/art.asp?articlekey=17762), impaired concentration, [somnolence](http://www.rxlist.com/script/main/art.asp?articlekey=13097), [abnormal](http://www.rxlist.com/script/main/art.asp?articlekey=22433) dreaming, [euphoria](http://www.rxlist.com/script/main/art.asp?articlekey=11351), confusion, agitation, [amnesia](http://www.rxlist.com/script/main/art.asp?articlekey=2223), hallucinations, stupor, abnormal thinking, and depersonalization. Serious psychiatric adverse experiences have been reported in patients treated with efavirenz. In controlled trials, the frequency of specific serious psychiatric symptoms among patients who received efavirenz or control regimens, respectively, were severe depression (2.4%, 0.9%), suicidal ideation (0.7%, 0.3%), nonfatal suicide attempts (0.5%, 0%), aggressive behavior (0.4%, 0.5%), paranoid reactions (0.4%, 0.3%) and maniac reactions (0.2%, 0.3%). Additional psychiatric symptoms observed at a frequency of >2% among patients treated with efavirenz or control regimens, respectively, in controlled clinical trials were depression (19%, 16%), anxiety (13%, 9%) and nervousness (7%, 2%). Rashes are usually mild-to-moderate maculopapular skin eruptions that occur within the first 2 weeks of initiating [therapy](http://www.rxlist.com/script/main/art.asp?articlekey=10897) with efavirenz. In most patients, rash resolves with continuing efavirenz therapy within one month. Efavirenz can be reinitiated in patients interrupting therapy because of rash. Use of appropriate [antihistamines](http://www.rxlist.com/script/main/art.asp?articlekey=2283) and/or corticosteroids may be considered when efavirenz is restarted. Efavirenz should be discontinued in patients developing severe rash associated with blistering, [desquamation](http://www.rxlist.com/script/main/art.asp?articlekey=6631), mucosal involvement, or [fever](http://www.rxlist.com/script/main/art.asp?articlekey=3425). The most common laboratory anomalies are liver function alterations, increase in lipids levels and false positive urine cannabinoid.

**7. CLINICAL AND LABORATORY ASSESSMENTS**

All clinical and laboratory evaluations should be performed at the scheduled time points and in case of patient’s worsening conditions or drug discontinuation.

**7.1 Screening/Baseline Evaluations**

- clinical evaluation;
- haematology and blood chemistry;
- immunology;
- virology.

**7.2 Clinical evaluation**

Clinical evaluation should be performed at baseline and after 4, 12 and 24 weeks.

Clinical evaluation will include medical history (age, gender, HIV infection risk factors, CDC stage, previous opportunistic infections) and physical examination, with performance status and body weight.

During the study the course of HIV-related clinical characteristics (fever, cough, body weight, night sweats) will be carefully monitored.

**7.3 Laboratory tests**

Laboratory tests will be performed at baseline and after 4, 12 and 24 weeks, including:

- CBC with differential and platelet count;
- serum chemistry: glucose, blood urea nitrogen, creatinine, sodium, potassium, chloride, carbonate, AST, ALT, CPK, total bilirubin, alcaline phosphatase, pancreatic amylases, total cholesterol, triglycerides, LDH, seroproteic electrophoresis, VES.

**7.4 Immunology**

The following immunological parameters will be evaluated at enrolment and after 4, 12 and 24 weeks:

**Lymphocyte immunephenotyping:** lymphocyte subsets will be evaluated by means of flow- cytometry, using 50 µl EDTA-treated peripheral blood incubated for 30 min at 4°C with flourochromes (fluorescein isothiocyanate [FITC], phycoerytrin [PE], and phycoerytrin-cyanin 5 [PCy5]). The following combinations of monoclonal antibodies will be used: CD45RA/CD62L/CD4, CD4/CD38, CD8/CD38 to measure naïve, memory and activated CD4+ and CD8+; T-cell proliferation degree will be measured through quantification of Ki67 nuclear antigen expression on CD4+ and CD8+ T cells, which is expressed in the proliferative phases of cell cycle, as previously described [16]. Maturation state of CD8+ lymphocytes will be established by evaluating the expression of CCR7/CD45RA and perforin.

**Evaluation of spontaneous apoptosis**: percentages of apoptotic CD4+ and CD8+ T-cells will be measured on fresh patient peripheral blood mononuclear cells (PBMC) separated from blood plasma by Ficoll-Hypaque (Sigma-Aldrich, Milan, Italy) using flow cytometry Annexin V/7-aminoactinomycin D staining.

**Thymic function analyisis**: thymic function will be measured through the quantification of “T-cell receptor excision circles” (TRECs) in peripheral blood CD4+ and CD8+ T lymphocytes, by using a recently described PCR-ELISA, that measures the “coding-joint” α TREC, which results from the recombinant events leading to the rearrangement of α gene after locus δ excision. Specifically,  to measure TRECs, CD4+ and CD8+ will be separated from PBMCs using magnetically beads coniugated with mAB anti-CD4 and anti-CD8 (CD4 and CD8 Positive isolation Kit, Oxoid, Milan, Italy) (purity > 95%). Briefly, after Ficoll separation of PBMC, CD4 and CD8 cells will be immediately immunomagnetically separated, pelleted, and frozen at -80°C. One and 0.5 μg DNA will be then extracted using DNAzol (Life Technologies, Milano, Italy) and stored until the next use. For every PCR-ELISA reaction, standard will be amplified in duplicate and OD median value will be used to quantify the number of “coding joint” VαJα TREC as copies/μg. Every clinical sample will be evaluate in triplicate.

TREC frequency will be measured as copies/µg and will be correlated to the cell turnover through the measure of Ki67. To further reduce the effect of cells proliferation on TREC, we will also calculate total TREC content, i.e., TREC/µl blood: (TREC/CD4) × (absolute number of CD4 cells), assuming that 1 µg genomic DNA equals approximately 150 000 cells.

**IL-7 and IL-15 plasma levels:** plasmatic levels of IL-7 and IL-15 will be quantify on thawed samples by using ELISA kits (Quantikine HS human IL-7 and Quantikine Human IL-15; R&D, Minneapolis, Minnesota, USA) following manufacturers' instructions.

**IL-7 and IL-15 production:** IL-7 production will be measured as recently described [17]; furthermore an assay for the quantification of IL-7 (GenBank, accession N° NM 000880) and IL-15 m-RNA by means of a real time PCR (TaqMan^TM^ RT-PCR) will be set up.

**IL-7Rα production:** a real time PCR (TaqMan^TM^ RT-PCR) assay will be set up for the quantification of IL-7Rα mRNA.

**IL-7Rα (CD127) expression on CD4+ and CD8+:** the expression of IL-7Rα on CD4+ and CD8+ T lymphocytes and on HIV-specific CD4+ and CD8+ T lymhpocytes will be measured using flow cytometry anti-CD127 (IL-7Rα) m-Ab. The proportion if CD8+IL-7R^high^ and CD8+IL-7R^low^ on CD8+ T lymphocytes will be measured through an approach from literature.

**HIV-specific CD4+ lymphocytes:** number, cytokine (IFN-γ and IL-2), production, maturation pattern (expression of CD45RA, CCR7), receptor expression (IL-2Rα or CD25, IL-15Rα) will be measured by flow-cytometry

**HIV-specific CD8+ lymphocytes**: number, IFN-γ and perforin production, maturation pattern (expression of CD45RA, CCR7, CD27), receptor expression (IL-2Rα or CD25, IL-15Rα) will be measured by flow-cytometry.

**Treg lymphocytes (CD4+CD25++):** Treg lymphocytes will be determined through anti-CD4, anti-CD25 mAbs: Treg lymphocytes are distinguishable by a constitutively increased expression of CD25, the α chain of IL-2R (CD25++ or bright). For the definition of CD25++ expression level, the citofluorimetric gate will be established by determining the cellular expression of CD122 (β subunit common to IL-2R and IL-15R) and the citoplasmatic presence of CD152 (Cytotoxic T lymphocyte-associated antigen 4, CTLA-4), as Treg lymphocytes are CD122++ and cCD152+. Number and grade of maturation (expression of CD62L) will be determined. Tregs will be further detected through the expression of Foxp3 gene with Real-Time PCR.

**7.5 Virology**

The following virologic parameters will be evaluated at enrolment and a7 Day 7, Day 15, Day 45, Week 4, Week 12, Week 24:

**Evaluation of the kinetics of HIV-RNA decay in plasma of patients.** HIV-RNA plasma levels will be measured using 2 different assays: a classical assay from the commerce with a lower limit of detection of 50 cp/ml, and an ultrasensitive assay with a detection limit of 5 cp/ml.

**Evaluation of the kinetics of intracellular HIV DNA in total peripheral blood CD4+.** For analysis of intracellular HIV DNA content, DNA will be extracted from total CD4+ T-cells, as previously described [18].

**8. DATA ANALYSIS**

Paired T test and Wilcoxon Signed-rank Test will be performed for normally and not normally distributed variables, respectively. All p-values produced will regarded as descriptive and no inferences will be made. Statistical analysis will be perfomed using EP16 software and SPSS software.

**9**. **WITHDRAWING FROM THE STUDY**

Cause and time of withdrawing from the trial must be reported on patient’s clinical record.

Subjects will be informed that their participation is voluntary and that they may withdraw from the trial at any time. They will be informed that choosing to withdraw from the trial will not have an impact on the care the subject will receive for the treatment of his/her disease. Moreover, subjects may be withdrawn from the trial if the investigator considers it is the best interest to the subject, for safety reason, that he or she be withdrawn.

**10. ETHICS**

**10.1 Declaration of Helsinki and ethics committee**

This study will be conducted according to the principles of the declaration of Helsinki. This trial can only be undertaken after full approval of the protocol, informed consent and any other written information given to the subjects, has been obtained from the Ethics Commitee. Investigator will take charge of notifying any serious adverse event to the Ethics Commitee. Substantial amendments must promptly be submitted to the Ethics Committee for review and approval prior to implementation of the change(s), except when necessary to eliminate an immediate hazard to the trial subjects.

**10.2 Subject information and informed consent**

Prior to entry in the trial, the investigator must explain to potential subjects the trial and the implications of participation. Moreover, he is responsible for the obtainment of written informed consent before study inclusion of the patient (see attached document).

**11. SOURCE DATA AND CASE REPORT FORMS**

The investigator shall maintain the trial documents and all sources of original data, including names and address of patients and signed informed consents, for 15 years.

For each patient a clinical record must be completed whose accuracy and reliability will be certified by investigator’s signature. Corrections must be made in such a way that the original entry is not obscured. Correction fluid must not be used.

The investigator will warrant that study participants have an appropriate training and that any relevant information could be communicate to the co-investigators and to the involved staff.

**REFERENCES**
