# Supplementary material for: Prospective Immune Dynamics during the First 24 Weeks of Efavirenz Based-Antiretroviral Therapy in HIV-1-Infected Subjects, According to CD4+ T-Cell Counts at Presentation: The IMMUNEF Clinical Trial
Source: PLoS One. 2015 Feb 11;10(2):e0117118. doi: 10.1371/journal.pone.0117118 (PMC4324909; doi:10.1371/journal.pone.0117118)
Supplement: S1 Table — (DOC) [file pone.0117118.s003.doc]

**Supplementary Table 1**. Baseline characteristics (categorical variables) of late and early presenters in the IMMUNEF study.

|  | **Late presenters**  **N = 13** | **Early presenters**  **N = 11** | **P-value** |
| --- | --- | --- | --- |
| Female gender, N (%) | 1 (7.7) | 2 (18.2) | 0.58 |
| Route of HIV acquisition, N (%)  Heterosexual  IVDU  MSM | 8 (61.5)  0 (0.0)  5 (38.5) | 5 (45.5)  1 (9.1)  5 (45.5) | 0.54 |
| HBV coinfection, N (%) | 2 (15.4) | 0 (0.0) | 0.48 |
| HCV coinfection, N (%) | 2 (15.4) | 1 (9.1) | 1.00 |
| Past AIDS-defining event, N (%) | 4 (30.8) | 0 (0.0) | 0.10 |

IVDU: intravenous drug users; MSM: men who have sex with men; HBV: hepatitis B virus; HCV: hepatitis C virus
